# Identification of epistatic interactions through genome-wide association studies in sporadic medullary and juvenile papillary thyroid carcinomas

**DOI:** 10.1186/s12920-015-0160-7

**Published:** 2015-12-21

**Authors:** Berta Luzón-Toro, Marta Bleda, Elena Navarro, Luz García-Alonso, Macarena Ruiz-Ferrer, Ignacio Medina, Marta Martín-Sánchez, Cristina Y. Gonzalez, Raquel M. Fernández, Ana Torroglosa, Guillermo Antiñolo, Joaquin Dopazo, Salud Borrego

**Affiliations:** Department of Genetics, Reproduction and Fetal Medicine, Institute of Biomedicine of Seville (IBIS), University Hospital Virgen del Rocío/CSIC/University of Seville, Seville, Spain; Centre for Biomedical Network Research on Rare Diseases (CIBERER), Seville, Spain; Computational Genomics Department, Centro de Investigación Príncipe Felipe (CIPF), C/Eduardo Primo Yúfera, 3, 46012 Valencia, Spain; Present Address: Department of Medicine, University of Cambridge, School of Clinical Medicine, Addenbrooke’s Hospital, Hills Road, Cambridge, UK; Department of Endocrinology, Institute of Biomedicine of Seville (IBIS), University Hospital Virgen del Rocío/CSIC/University of Seville, Seville, Spain; Present Address: European Bioinformatics Institute (EMBL-EBI), European Molecular Biology Laboratory, Wellcome Trust Genome Campus, Hinxton Cambridge, UK; Present Address: HPC Services, University of Cambridge, Cambridge, UK; Functional Genomics Node, (INB) at CIPF, Valencia, Spain

**Keywords:** Sporadic medullary thyroid carcinoma, Juvenile papillary thyroid carcinoma, Epistasis, Multifactor-dimensionality reduction, Genome-wide association study

## Abstract

**Background:**

The molecular mechanisms leading to sporadic medullary thyroid carcinoma (sMTC) and juvenile papillary thyroid carcinoma (PTC), two rare tumours of the thyroid gland, remain poorly understood. Genetic studies on thyroid carcinomas have been conducted, although just a few loci have been systematically associated. Given the difficulties to obtain single-loci associations, this work expands its scope to the study of epistatic interactions that could help to understand the genetic architecture of complex diseases and explain new heritable components of genetic risk.

**Methods:**

We carried out the first screening for epistasis by Multifactor-Dimensionality Reduction (MDR) in genome-wide association study (GWAS) on sMTC and juvenile PTC, to identify the potential simultaneous involvement of pairs of variants in the disease.

**Results:**

We have identified two significant epistatic gene interactions in sMTC (*CHFR-AC016582.2* and *C8orf37-RNU1-55P*) and three in juvenile PTC (*RP11-648k4.2-DIO1, RP11-648k4.2-DMGDH* and *RP11-648k4.2-LOXL1*). Interestingly, each interacting gene pair included a non-coding RNA, providing thus support to the relevance that these elements are increasingly gaining to explain carcinoma development and progression.

**Conclusions:**

Overall, this study contributes to the understanding of the genetic basis of thyroid carcinoma susceptibility in two different case scenarios such as sMTC and juvenile PTC.

**Electronic supplementary material:**

The online version of this article (doi:10.1186/s12920-015-0160-7) contains supplementary material, which is available to authorized users.

## Background

Thyroid carcinoma is the most common endocrine malignancy, which account for more than 1 % of all new malignant tumors [1]. There are several histological types and subtypes according to the endocrine thyroid cells from which thyroid carcinomas are derived. Medullary thyroid carcinoma (MTC) arises from calcitonin-producing parafollicular cells (thyroid C cells) and constitutes around 2–5 % of all thyroid neoplasias [2]. Approximately 25 % of MTC cases present an autosomal dominant inherited disorder named Multiple Endocrine Neoplasia type 2 (MEN 2), which includes three different clinical phenotypes: MEN 2A, MEN 2B, and familial MTC (FMTC) [3]. In >95 % of the cases, the three forms of MEN 2 are caused by specific gain-of-function germline mutations of the *RET* proto-oncogene [3, 4]. The remaining 75 % of MTC occurs as a non-inherited sporadic lesion without associated endocrinopathy (sporadic MTC, sMTC). Unlike hereditary forms, very little is known about the genetic basis of sMTC. So far, studies have been focused on the analysis of specific single nucleotide polymorphisms (SNPs)/haplotypes and significant associations to sMTC have been described for different susceptibility genes, suggesting that this phenotype may be caused by a combination of multiple common genetic variants [5–7]. However, most of them have failed to be replicated in other populations, maybe due to the difficulty in collect enough patients to reach the necessary statistical power [6].

Nonmedullary thyroid carcinoma (NMTC) comprises thyroid carcinomas of follicular cell origin and among them papillary thyroid carcinoma (PTC) and follicular thyroid carcinoma represent the two most common subtypes (85 and 10 %, respectively) [8]. Regarding PTC, the age is considered as one of the most important prognostic factors. Juvenile PTC (jPCT), a rare disease in children and adolescents, presents with an aggressive initial manifestation, though patients have an excellent overall prognosis, showing longer periods of survival and a lower incidence of recurrence [9, 10]. However, PTC remains under-reported in children and adolescents and there is still disagreement about the standard treatment and optimal type of follow-up needed [9, 11]. The few pathologic studies carried out in jPTC point to exposure to ionizing radiation as the only known environmental risk factor [12, 13]. Although it have reported several SNPs associated with the risk of PTC in the absence of radiation, most of them were carried out with older patients (mean age around 45 years) and/or using somatic rather than germline DNA [6]. As in the case of sMTC, the molecular mechanisms that account for jPTC or explain the development of this tumour remain largely unknown.

Despite the success of GWAS [14] in the identification of hundreds of genetic variants associated to different diseases [15], its application to rare and multifactorial diseases still presents major drawbacks. Conventional GWAS approaches, based on single markers, require of large cohorts, typically unavailable in rare diseases. In fact, the prevalence of sMTC is approximately 7/100,000, according to Orphanet (see http://www.orpha.net/orphacom/cahiers/docs/GB/Prevalence_of_rare_diseases_by_alphabetical_list.pdf) and there are no data available for PTC. Additionally, the complex genetic nature of some diseases make single marker approaches quite inefficient, while suggest taking approaches based on systems biology [16].

We carried out a GWAS in two thyroid carcinoma types (sMTC and jPTC) followed by the detection of epistatic interactions to identify the potential simultaneous involvement of pairs of variants in both diseases. The determination of genome-wide epistasis encompasses statistical and computational challenges [17]. Here, we have used the Multifactor-dimensionality reduction (MDR) method to detect gene-gene interactions (GxG) in GWAS, which has already successfully been used in the detection of multiple and joint genetic factors associated with complex traits [18].

We report here the first study that combines a screening for epistasis by MDR in GWAS on these two rare thyroid carcinoma types.

## Methods

### Patients and controls

Two different series of Spanish patients were recruited for this study: one with 66 sMTC patients, with absence of personal or family history suggestive of MEN 2 and absence of traditional germline MEN 2-defining *RET* mutations and another with 38 jPTC (range from 5 to 24 years of age) with no history of head and/or neck irradiation (see Additional file 1: Table S1) and no familiar aggregation. Additionally, 129 healthy controls comprising unselected, unrelated, race, age, and sex-matched individuals without previous thyroid-related disease history were recruited and used as controls of both carcinoma types.

All subjects underwent peripheral blood extraction for genomic DNA isolation using MagNA Pure LC system (Roche, Indianapolis, IN) according to the manufacturer’s instructions.

A written informed consent was obtained from all the participants for clinical and molecular genetic studies. The study was approved by the Ethics Committee for clinical research in the University Hospital Virgen del Rocío (Seville, Spain) and complies with the tenets of the declaration of Helsinki.

### Genome-wide genotyping

DNA derived from peripheral blood was hybridized to Affymetrix Genome-Wide Human SNP 6.0 arrays. CEL files were processed using Affymetrix Power Tools (APT v1.15.0) and genotypes were obtained using the Birdseed (v2) calling algorithm.

### Data quality control

Quality control was carried out using PLINK software [19]. Individuals with more than 7 % of missing rate as well as those with excessive or reduced heterozygosity (+/-2 times the standard deviation of the mean) were removed. The data were also checked for duplicates and related individuals. Finally, divergent ancestry was checked using EIGENSTRAT [20] to detect possible population stratification.

Markers with excessive missing rate (>5 %) were discarded. SNPs with minor allele frequency < 1 % and/or not in Hardy-Weinberg equilibrium (in unaffected samples; *P* < 1 × 10^−5^) were also excluded.

The data discussed in this publication have been deposited in NCBI’s Gene Expression Omnibus [GEO:GSE67047].

### Association and gene-gene interaction tests

We carried out a conventional GWAS in our series of patients of jPTC and sMTC. In the analysis strategy followed we have used a Quantile-quantile plot (QQplot) to decide the most appropriate measures of association and relevance of disease models as well as the most suitable test of association [21]. Thus, QQplots and Manhattan plots were generated for each of the models to assess their validity. PLINK permits the GWAS analysis using two different approaches. Models were adjusted by sex and age and no significant differences were found with the unadjusted models. For the best fit model, the final *p*-value estimations were corrected using the Benjamini and Hochberg’s FDR adjustment [22].

We have reduced the number of SNPs to those representatives of genomic blocks in linkage disequilibrium by searching for tag SNPs using the Tagger software [23]. The study of possible epistatic interactions between pairs of genes was carried out with the Multifactor-dimensionality reduction (MDR) method [18]. MDR allows reducing the dimensionality of SNP data improving the identification of combinations of polymorphisms associated with disease risk. MDR is nonparametric, model-free and can directly be applied to case–control [18]. MDR implements a 10-fold cross-validation value (CVV) which is taken as an indicator of detection of pairs of SNPs showing an association to the disease significantly over the random expectation. Only tag SNPs mapping in genes were used in this study. In addition to coding genes we included in the study any ncRNA and pseudogenes. Despite the dimensionality reduction produced by using tag SNPs, the study of all the interactions still constitutes a challenge. Then, a parallelized version of the MDR method was developed. Our implementation uses all cores in a machine and also distributes the work among the nodes in a cluster. The multi-core part has been implemented using OpenMP directives and SSE instructions. Task distribution across processors is managed by the MPI applications programming interface [24]. The software is open and can be found at https://github.com/opencb/variant.

The main SNPs on the relevant interacting genes detected in sMTC and in jPTC were validated by Taqman technology using 7900HT Fast Real-Time PCR System (Applied Bio- systems, Foster City, California, USA)

### Functional interpretation of the results

Functional evidences for the identified epistatic gene interactions were exhaustively analysed using a number of available annotation repositories and functional analysis tools. Gene functionality was assessed using the following resources: *DAVID v6.7* [25], *canSAR 2.0* [26], and *COSMIC* [27] databases. Gene-phenotype relationships were explored with *VarElect* (phenotype assignation tool from *GeneCards*) [28] and *GeneMANIA* [29]. Information on gene expression localization was taken from the *Human Protein Atlas* [30] and the *GEO* repository [31]. Finally, information about non-coding RNAs was obtained from *neXtProt* [32], *NONCODE v4.0* [33] and Ensembl through the *CellBase* application [34].

## Results and discussion

After the quality control process, a total of 158 samples in the sMTC vs control dataset (49 sMTC and 109 control with 639,289 SNPs) and 149 samples in the PTC *vs* control dataset (38 PTC and 111 control with 640,597 SNPs) remained (Additional file 1: Tables S2 and S3). After the application of the Tagger software a total of 357,263 tag SNPs in MTC and 344,455 in PTC remained.

In the last decade, many studies on thyroid carcinomas have been conducted but only a few loci have been systematically associated to the disease. The study of epistatic interactions could overcome some of the limitations of the conventional GWAS studies, helping to understand the genetic architecture of the disease and explain new heritable components of genetic risk [16].

### Individual SNP associations to the diseases

The results of the conventional GWAS analysis render no significant associations (after multiple testing adjustments) for jPTC and only 21 SNPs with marginally significant associations for MTC (see Additional file 1: Table S4).

Among these SNPs, 6 appear in intergenic regions. The rest of them map in genes or around genes, although, none of them was previously associated to MTC [6]. This is expectable given that most of the known associations are gene or pathway-driven associations and have thus more statistical power, although less reproducibility [6].

### Epistastic interactions in sMTC

**Fig. 1:**
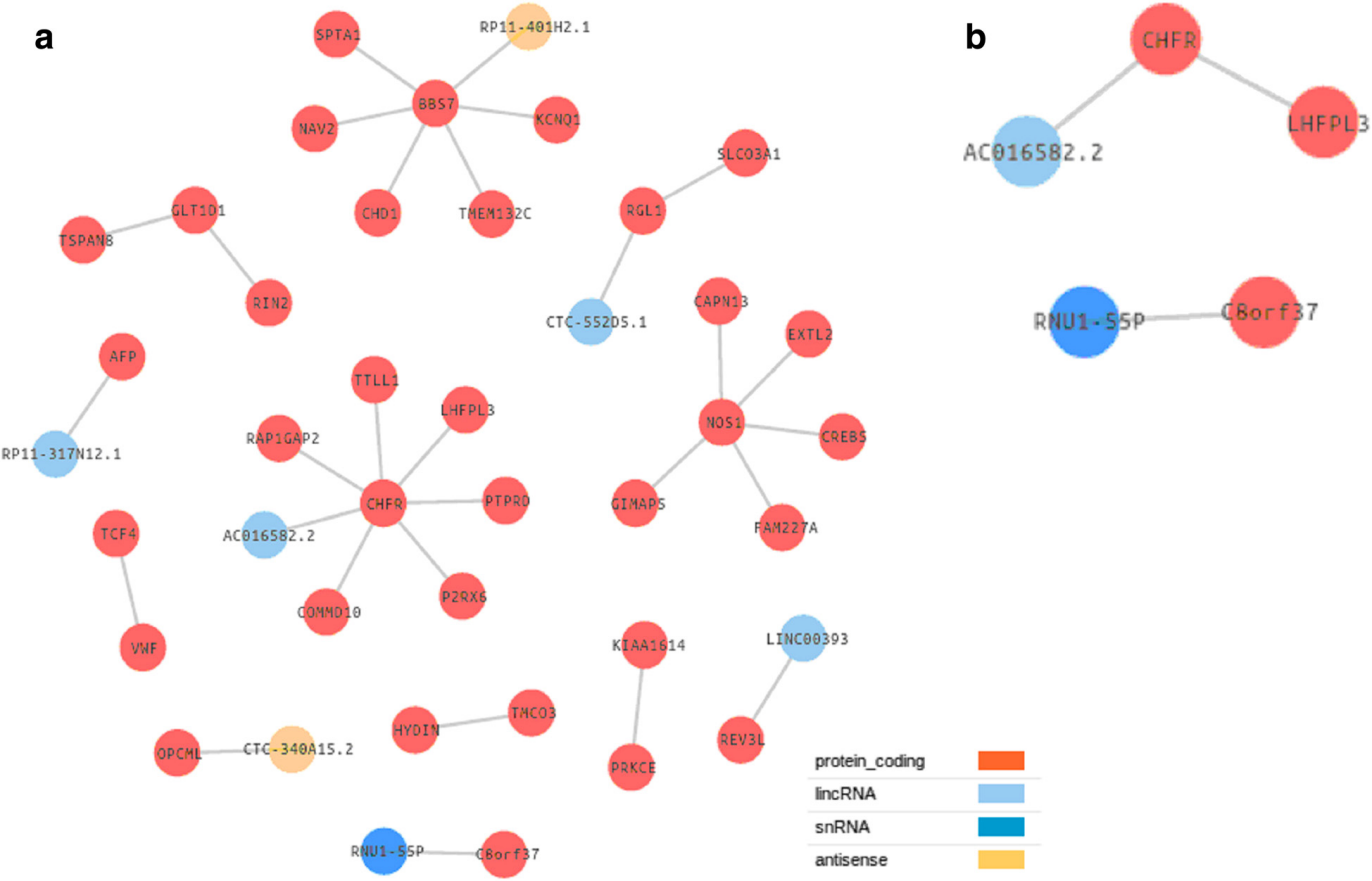
Gene-gene interactions obtained in sMTC patients by MDR analyses. **a** a total of 29 GxG interactions were obtained, (**b**) from which three were significant based on cross-validation value (>0.5)

Epistatic analysis of the sMTC samples genotypes (Table 1; Fig. 1) by MDR revealed three gene-gene interactions (*LHFPL3-CHFR*, *CHFR-AC016582.2* and *C8orf37-RNU1-55P*) significantly associated with the disease (CVV > 0.5). Here we suggest *CHFR- AC016582.2* (rs4758915, adjusted *p*-value = 0.9812, and rs10402530, adj. *p*-value = 0.9936, respectively) and *C8orf37-RNU1-55P* (rs7835921, adj. *p*-value 00.9669, and rs1287079, adj. *p*-value = 0.9569, respectively) as interesting potential candidate genes in sMTC. Table 2 contains the *p*-values as well as ORs and frequencies in cases and controls observed for the SNPs. Next, these SNPs were successfully validated by Taqman technology. It is more unlikely that *LHFPL3* plays a relevant role in thyroid gland given that it is not expressed according to the databases of gene expression (GEO ID: GDS1665, corresponding to “Papillary thyroid carcinoma”). This does not mean that this gene cannot be a candidate, given that the expression of cancer-related genes in total thyroid tissue could be minority and hence undetectable in the databases, but here we are focusing on the most likely candidates.

**Table 1:** Gene-gene significant interactions in sMTC obtained by MDR

SNP 1	SNP 2	Gene 1	Gene 2	Gene name 1	Gene name 2	CVV
rs7787988	rs4758915	ENSG00000187416	ENSG00000072609	*LHFPL3*	*CHFR*	0.709
rs4758915	rs10402530	ENSG00000072609	ENSG00000225868	*CHFR*	*AC016582.2*	0.706
rs7835921	rs1287079	ENSG00000156172	ENSG00000202380	*C8orf37*	*RNU1-55P*	0.548

Selection based on the significant cross-validation value (CVV)

**Table 2:** SNPs involved in significant interactions. Columns from left to right are: SNP identifier, region in which the SNP is located, gene, Frequencies in the cases, frequencies in the controls, ORs and 95 % of confidence interval (CI), nominal *p*-value and adjusted *p*-value

Analysis	SNP	Region	Gene	Frequency cases	Frequency controls	OR [95 % CI]	*P*-value	Adjusted *p*-value
sMTC	rs4758915	Intronic	CHFR	0.03704	0.02586	1.449 [0.4003–5.243]	0.5702	0.9812
sMTC	rs10402530	Downstream	AC016582.2	0.1944	0.1897	1.031 [0.5782–1.84]	0.9167	0.9936
sMTC	rs7835921	Intronic	C8orf37	0.4519	0.5086	0.7966 [0.5008–1.267]	0.3365	0.9669
sMTC	rs1287079	Downstream	RNU1-55P	0.3019	0.3664	0.7479 [0.4567–1.225]	0.2476	0.9569
jPTC	rs2235544	Intronic	DIO1	0.4667	0.5043	0.86 [0.487–1.519]	0.6032	0.9696
jPTC	rs16876356	Intronic	DMGDH	0.1167	0.2308	0.4403 [0.1891–1.025]	0.0518	0.9007
jPTC	rs17716031	Intronic	RP11-648K4.2	0.1034	0.1336	0.7481 [0.2964–1.888]	0.5379	0.9668
jPTC	rs10775207	Intronic	LOXL1	0.0167	0.0087	1.932 [0.1723–21.67]	0.5868	0.9668

In particular, *CHFR* gene encodes an E3 ubiquitin-protein ligase that acts in the mitotic checkpoint and functions as a tumour suppressor [35]. *CHFR* gene displays a significant epistatic association with *AC016582.2,* which is a novel long intergenic non-coding RNA (lincRNA). To date, there are no studies that have linked lincRNAs to sMTC yet.

Regarding *C8orf37* gene, it encodes a ubiquitously expressed protein of unknown function. This gene has found to be over-expressed in astrocytoma, prostate carcinoma and undifferentiated sarcoma, as well as under-expressed in benign prostate hyperplasia (*canSAR* database). Moreover, copy number variation including this gene has been described in a thyroid carcinoma cell line (Human Protein Atlas database). *C8orf37* gene was found in epistasis with *RNU1-55P* (RNA, U1, small nuclear 55, pseudogene). Small nuclear RNAs (snRNA) are known to act as guide molecules for pseudouridylation and site-specific methylation of other RNAs [36]. For example, a novel snRNA (RN7SK) has been described to interact with *HMGA1* gene, which is overexpressed in thyroid carcinoma [37]. *RNU1-55P* is also a pseudogene, which can play an important role in physiology and disease (e.g. as *BRAF* pseudogene in different thyroid carcinomas [38]). It is noteworthy that one of the genes implicated in each of the two relevant pairs of gene interactions in sMTC was a type of ncRNA.

### Epistastic interactions in jPTC

**Fig. 2:**
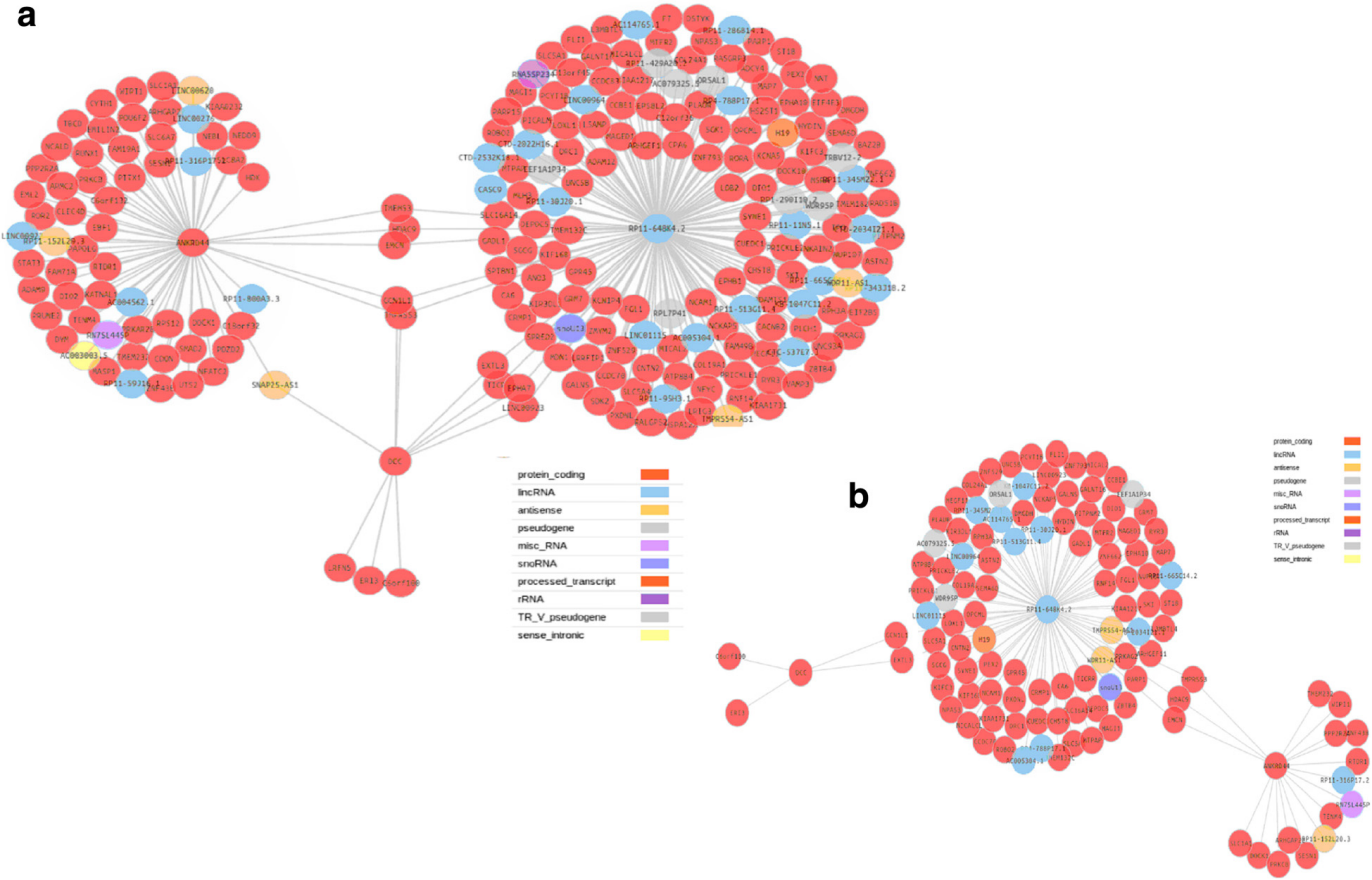
Gene-gene interactions obtained in jPTC patients by MDR analyses. **a** a total of 259 GxG interactions were obtained, (**b**) from which 133 were significant based on cross-validation value (>0.5)

**Fig. 3:**
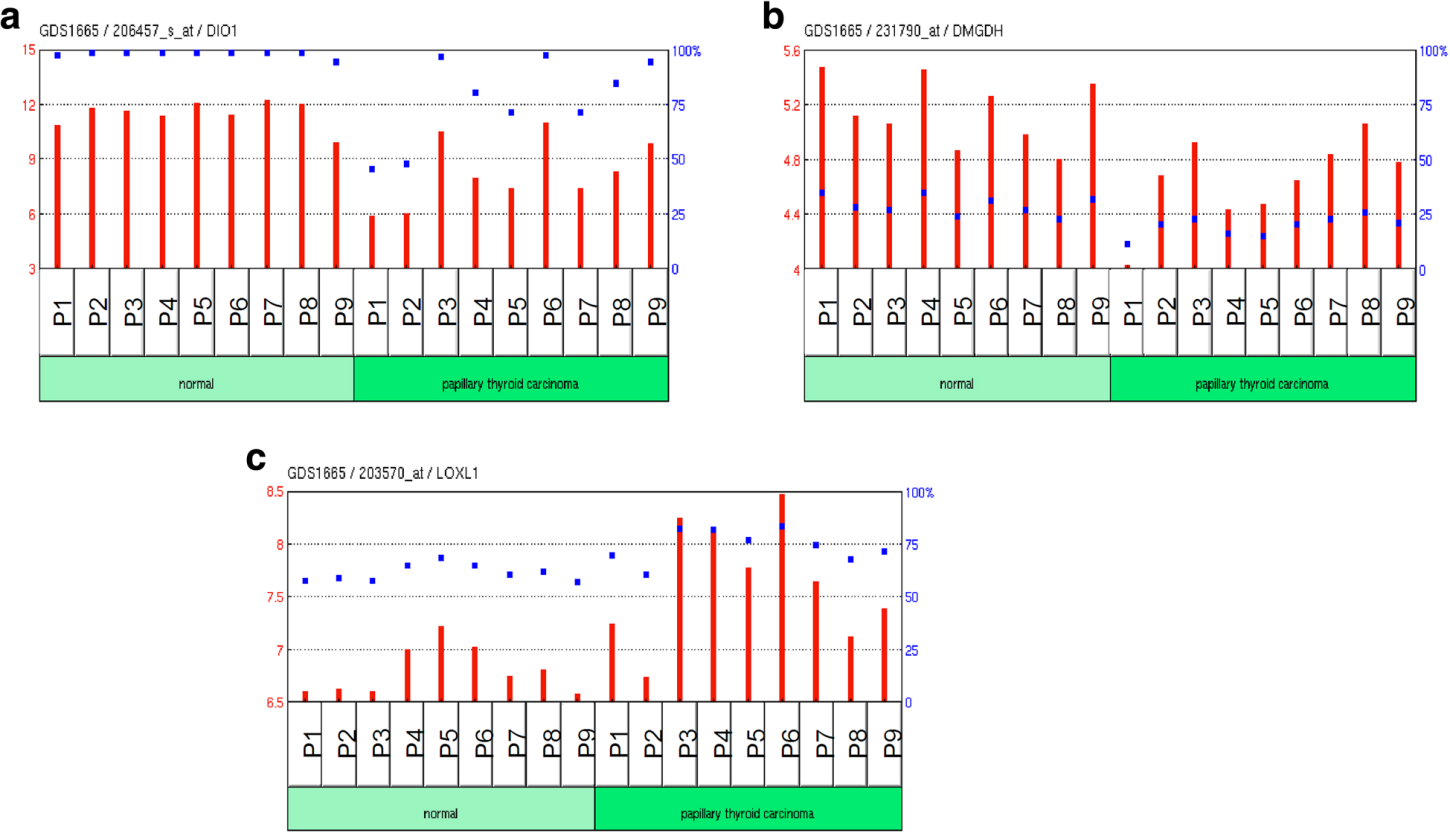
Expression profiling for arrays in 18 PTC samples (**a**) *DIO1,* (**b**) *DMGD* and (**c**) *LOXL1* genes. Figures adapted from GeoDataSets

MDR analysis of jPTC samples (Additional file 1: Table S5) rendered a total of 133 GxG interactions related with the disease (with a CVV >0.5) (Fig. 2). Among all these interactions, we focused on those more likely to be related to the disease, according to their functionalities and gene expression patterns. The application of the DAVID and Babelomics [39] tools resulted in several gene clusters, one of them composed of 10 genes associated with thyroid gland. According to the CVV values obtained by MDR analyses, only five of those 10 genes were significantly associated with the phenotype (*CHST8, DIO1, DMGDH, LOXL1* and *PXDNL*). Among these genes, *DIO1*, in epistasis with *RP11-648k4.2*, was directly associated to the disease by the VarElect genotype-phenotype association tool (Tables 3, 4 and Fig. 3). Available information on gene expression in GEO allowed us to discard *CHST8* and *PXDNL* genes because no difference among normal and pathologic tissue expression was reported for them, while both *DIO1* and *DMGDH* genes were underexpressed and *LOXL1* gene was overexpressed in thyroid carcinoma [31]. In particular, *DIO1* encodes an iodothyronine deiodinase type 1, which is a protein linked to carcinoma risk and specifically with PTC [40]. This link between *DIO1* and PTC reinforces the validity of epistasis as an optimal strategy to find candidate genes for rare diseases or, in general, when large sample sizes are not available.

**Table 3:** Direct and indirect gene-gene interactions obtained by GWAS-epistasis analyses in jPTC patients by VarElect

Direct GxG interactions					
Significant interaction			Not significant interaction		
Gene	Gene	CVV	Gene	Gene	CVV
*RP11-648k4,2*	*DIO1*	0.559	*ANKRD44*	*STAT3*	0.280
			*RP11-648k4,2*	*TPO*	0.371
Indirect and significant interactions					
Gene	Gene	CVV	Gene	Gene	CVV
*RP11-648K4.2*	*ARHGAP22*	0.559	*RP11-648k4,2*	*PCYT1B*	0.650
	*ARHGEF11*	0.555		*PLAUR*	0.851
	*ATP8B4*	0.748		*PRICKLE1*	0.557
	*CA6*	0.744		*PRICKLE2*	0.744
	*CHST8*	0.555		*PRKAG2*	0.556
	*CNTN2*	0.557		*RNF14*	0.650
	*COL19A1*	0.651/0.555		*ROBO2*	0.646
	*COL24A1*	0.747		*RYR3*	0.559
	*CRMP1*	0.744		*SEMA6D*	0.558
	*DMGDH*	0.747		*SGCG*	0.557
	*EXTL3*	0.747		*SKI*	0.557
	*FLI1*	0.649		*SLC5A1*	0.654
	*GALNS*	0.650		*SLC5A4*	0.652
	*GALNT16*	0.555/0.555		*SYNE1*	0.650
	*GCN1L1*	0.650/0.650		*TICRR*	0.844
	*GPR45*	0.555		*UNC5B*	0.744
	*GRM7*	0.740		*ZNF529*	0.650
	*H19*	0.556		*ZNF662*	0.653
	*HDAC9*	0.649		*ZNF793*	0.653
	*KIFC3*	0.937	*ANKRD44*	*DOCK1*	0.652
	*KIR3DL1*	0.744		*HDAC9*	0.652
	*LOXL1*	0.743		*PPP2R2A*	0.652
	*MAGED1*	0.649		*PRKCB*	0.558
	*MAGI1*	0.650		*SESN1*	0.652
	*NCAM1*	0.650		*SLC1A1*	0.558
	*NPAS3*	0.652		*WIPI1*	0.942
	*NUP107*	0.649	*DCC*	*C6orf100*	0.651
	*OPCML*	0.743		*ERI3*	0.937
	*OR5AL1*	0.744		*EXTL3*	0.748
	*PARP1*	0.950/0.650/0.650		*GCN1L1*	0.558

More than one value in cross-validation value (CVV) cells means more than one interaction between these genes

**Table 4:** Analyses by DAVID and GeneMANIA of the five significant genes found in jPTC by MDR

		*CHST8*	*DIO1*	*DMGDH*	*LOXL1*	*PXDNL*
MDR Analyses		*RP11-648k4.2*	*RP11-648k4.2*	*RP11-648k4.2*	*RP11-648k4.2*	*RP11-648k4.2*
Interacting genes according to scientific literature		*-*	*DIO2*	*-*	*-*	*-*
Interacting genes according to DAVID		*HS2ST1, TMEM132C, TMEM182*	*-*	*-*	*-*	*-*
Interactions Analyses by GeneMANIA	Co-expression	*-*	*TPO, PXDNL, DMGDH*	*DIO1*	*-*	*DIO1*
	Co-localization	*-*	*TPO, DIO2*	*-*	*-*	*-*
	Genetic interactions	*ANKRD44, NNT*	*NNT*	*-*	*ANKRD44, TPO*	*-*
	Shared protein domain	*HS2ST1*	*DIO2*	*-*	*-*	*TPO*

Regarding *DMGDH* gene, it encodes a mitochondrial dimethylglycine dehydrogenase related with oxidative demethylation of dimethylglycine in vitro with the formation of sarcosine, hydrogen peroxide and formaldehyde [41]. *LOXL1* gene encodes a lysyl oxidase-like 1 enzyme involved in the connective tissue biogenesis. It has been related with different tumour progression and metastasis [42]. *LOXL1* gene is expressed in thyroid tissue and one registered mutation has been reported in this gene for PTC, although it remains to be validated (COSMIC database).

Thereby, we suggest *DIO1* (rs2235544, adj. *p*-value = 0.9696), *DMGDH* (rs16876356, adj. *p*-value = 0.9007) and *LOXL1* (rs10775207, adj. *p*-value = 0.9668) genes, all of them in epistasis with *RP11-648k4.2* (rs17716031, adj. *p*-value = 0.9668), a lincRNA which has been scarcely studied, as candidate genes for jPTC. See also ORs and frequencies in cases and controls observed for the SNPs in Table 2. Again, these SNPs were validated using Taqman technology. It is worth noting that different lincRNA have recently been linked to PTC. For example, a novel lincRNA gene (*PTCSC2*) has been found down-regulated in PTC tumours [43]. Also, up-regulation of a *BRAF*-activated lincRNA, previously associated with melanoma, has been related to PTC by increasing cell proliferation and autophagy activation [44]. It is important to highlight that these three genes, with a clear relationship with the PTC phenotype, found in jPTC were in epistasis with the same lincRNA *RP11-648k4.2*, which was also present in 181 additional genetic interactions, suggesting the potential involvement of this LincRNA in PTC. The large number of interactions displayed by *RP11-648k4.*2 suggests a regulatory role for this lincRNA. Our observation reinforces the increasingly important role of ncRNAs in carcinoma.

Given that both thyroid carcinomas are extremely rare, all the samples were used in this study and, consequently an independent population to validate the findings is not available. Future research with other cohorts will reveal whether our findings are generalizable to other populations or, on the contrary, different populations will display different susceptibility variants associated, that probably represent different facets of the mechanism of the disease, as it has been observed in other diseases [45].

## Conclusions

Here we present the first genome-wide screening to detect epistasis in two thyroid carcinoma entities as sMTC and jPTC. Among our results, we remark the significance of two epistatic interactions in sMTC and three in jPTC. In addition, it is worth mentioning the presence of ncRNAs, and especially lincRNAs, among the epistasis found. Such elements are acquiring an increasingly relevance in carcinoma research in recent years. Although further studies would be needed to corroborate the interactions found, our methodological approach has demonstrated to be a promising complementary tool for finding new susceptibility genes in these thyroid carcinomas.

## Additional file

Additional file 1: Tables S1 to S5. (DOC 257 kb)

## Abbreviations

CVV: Cross-validation value
FDR: False discovery rate
GWAS: Genome-wide association study
jPTC: Juvenile papillary thyroid carcinoma
MDR: Multifactor-dimensionality reduction
MEN 2: Multiple endocrine neoplasia type 2
NMTC: Nonmedullary thyroid carcinoma
OR: Odd ratio
PTC: Papillary thyroid carcinoma
sMTC: Sporadic medullary thyroid carcinoma
SNP: Single nucleotide polymorphism
