# Supplementary material for: Identification of epistatic interactions through genome-wide association studies in sporadic medullary and juvenile papillary thyroid carcinomas
Source: BMC Med Genomics. 2015 Dec 21;8:83. doi: 10.1186/s12920-015-0160-7 (PMC4685628; doi:10.1186/s12920-015-0160-7)
Supplement: Additional file 1: Tables S1 to S5. — (DOC 257 kb) [file 12920_2015_160_MOESM1_ESM.doc]

**Additional file 1: Table S1. Clinico-pathological data from jPTC patients included in the study.**

|  | | | |  |
| --- | --- | --- | --- | --- |
| **Patient** | **Age at diagnosis (years)** | **Sex** | **Extrathyroidal extension** | **Primary tumor /Lymph node metastases / Tumor node metastasis stage** |
| 1 | 20 | Female | No | TxN1M1 (Stage II) |
| 2 | 18 | Female | No | T1N1M0 (Stage I) |
| 3 | 5 | Female | No | TxN1M1 (Stage II). Persistent disease. |
| 4 | 12 | Female | Yes | TxN1M1 (Stage II) |
| 5 | 20 | Female | NA | TxN1M1 (Stage II) |
| 6 | 17 | Female | No | TxN1M0 |
| 7 | 24 | Female | No | T1N0M0 (Stage I) |
| 8 | 12 | Male | Yes | T4N1M1 (Stage II) |
| 9 | 11 | Female | No | TxN1M1 (Stage II) |
| 10 | 5 | Male | NA | TxN1M1 (Stage II) |
| 11 | 23 | Male | No | T2N1M1 (Stage II) |
| 12 | 19 | Male | Yes | T3N1M1 (Stage II) |
| 13 | 13 | Female | No | TxN1M0 (Stage I) |
| 14 | 15 | Female | No | T2N1M0 |
| 15 | 19 | Female | No | T1N1M1 (Stage II) |
| 16 | 19 | Female | No | T3N0M0 (Stage I) |
| 17 | 11 | Female | Yes | T4N1M1 (Stage II) |
| 18 | 19 | Female | No | T1N0M0 (Stage I) |
| 19 | 20 | Female | NA | TxN1M0 |
| 20 | 13 | Female | NA | TxN1M0 |
| 21 | 12 | Female | Yes | T3N1Mx |
| 22 | 12 | Female | No | T1N1M0 (Stage I) |
| 23 | 14 | Female | Yes | T4N1MX (Stage I) |
| 24 | 10 | Male | Yes | TxN1M1 |
| 25 | 15 | Female | No | T2N1M0 |
| 26 | 10 | Female | Yes | TxN1M1 |
| 27 | 19 | Female | NA | NA |
| 28 | 19 | Female | No | TxN0M0 |
| 29 | 18 | Female | Yes | T4N1M0 |
| 30 | 15 | Male | Yes | T2N1M0 (Stage I) |
| 31 | 19 | Male | Yes | TxN1M1 (Stage 2) |
| 32 | 16 | Male | Yes | T4N1M0 (Stage1) |
| 33 | 12 | Female | No | TxNxM1 (Stage 2) |
| 34 | 10 | Female | Yes | T3N1M0 (Stage I) |
| 35 | 18 | Female | Yes | T4N1M1 (Stage II) |
| 36 | 18 | Male | No | T2N0M0 (StageI) |
| 37 | 16 | Female | Yes | T3N0M0 (Stage I) |
| 38 | 11 | Female | No | TxN1M0 |

**Additional file 1: Table S2.** Number of samples excluded per dataset. Individuals with more than 7% of missing values, heterozygosity rates over 2 standard deviations of the mean, or Identity by Descent (IBD) over 0.185 were discarded from the study.

|  | MTC vs Control | | PTC vs Control | |
| --- | --- | --- | --- | --- |
|  | **MTC** | **Control** | **PTC** | **Control** |
| Elevated missing (>7%) or outlying heterozygosity rate (±2sd) | 7 | 6 | 0 | 6 |
| Related individuals (IBD > 0.185) | 4 | 6 | 0 | 6 |
| Divergent ancestry | 6 | 8 | 0 | 6 |

**Additional file 1: Table S3.** Number of SNPs excluded in every step of the QC.

|  | MTC vs Control | PTC vs Control |
| --- | --- | --- |
| Excessive missing rate (>5%) | 141541 | 148760 |
| Different genotype call rates between cases and controls | 384 | 3368 |
| Minor Allele Frequency (<1%) | 130050 | 121355 |
| HWE (*P* < 1 x 10^-5^) | 22035 | 22571 |

**Additional file 1: Table S4**. SNPs associated to MTC obtained by conventional GWAS association analysis.

| **SNP** | **Chr** | **Position** | **Region type** | **GENE** | **MAF Cases** | **MAF Controls** | **OR [95% CI]** | **P-value** | **Adj. p-value** |
| --- | --- | --- | --- | --- | --- | --- | --- | --- | --- |
| rs16878633 | 5 | 60504602 | nc_transcript_variant | CTC-436P18.1,  CTC-436P18.3 | 0.2115 | 0.02232 | 11.75 [4.307-32.06] | 5.60E-09 | 0.00358236 |
| rs10243320 | 7 | 3072230 | intron_variant | CARD11 | 0.1321 | 0.004386 | 34.54 [4.477-266.5] | 7.77E-08 | 0.02483806 |
| rs16862992 | 2 | 15885079 | downstream_gene_variant | AC008271.1 | 0.1538 | 0.01339 | 13.39 [3.808-47.11] | 1.69E-07 | 0.0304283 |
| rs35394679 | 5 | 98222262 | nc_transcript_variant | CHD1,  RNU6-402P | 0.1604 | 0.01739 | 10.79 [3.534-32.96] | 1.91E-07 | 0.0304283 |
| rs6049295 | 20 | 23979706 | intergenic_variant |  | 0.1635 | 0.01802 | 10.65 [3.484-32.55] | 2.38E-07 | 0.0304283 |
| rs2706801 | 4 | 122797512 | downstream_gene_variant | TRPC3,  RP11-63B13.1 | 0.1827 | 0.02273 | 9.612 [3.478-26.57] | 3.27E-07 | 0.03479651 |
| rs13897 | 11 | 66331169 | nc_transcript_variant | CTSF,ACTN3,  CTD-3074O7.2 | 0.1875 | 0.0307 | 7.286 [2.932-18.11] | 4.53E-07 | 0.03832136 |
| rs3731055 | 3 | 14220439 | intron_variant | XPC,LSM3 | 0.1415 | 0.01304 | 12.47 [3.527-44.11] | 5.63E-07 | 0.03832136 |
| rs7581924 | 2 | 180482000 | nc_transcript_variant | ZNF385B | 0.3723 | 0.1435 | 3.541 [2.028-6.184] | 6.00E-07 | 0.03832136 |
| rs9954705 | 18 | 76168940 | intergenic_variant |  | 0.1538 | 0.0177 | 10.09 [3.282-31.02] | 6.40E-07 | 0.03832136 |
| rs11826829 | 11 | 24794260 | nc_transcript_variant | LUZP2 | 0.1827 | 0.02655 | 8.196 [3.165-21.22] | 6.74E-07 | 0.03832136 |
| rs6695815 | 1 | 158636864 | intron_variant | SPTA1 | 0.1509 | 0.01754 | 9.956 [3.24-30.59] | 7.71E-07 | 0.03832136 |
| rs10492001 | 12 | 42394637 | intergenic_variant |  | 0.1415 | 0.01339 | 12.14 [3.433-42.95] | 8.23E-07 | 0.03832136 |
| rs7550036 | 1 | 173682365 | upstream_gene_variant | KLHL20 | 0.1759 | 0.03017 | 6.862 [2.788-16.89] | 8.82E-07 | 0.03832136 |
| rs7176836 | 15 | 29508833 | intron_variant | FAM189A1 | 0.1275 | 0.008772 | 16.51 [3.651- 74.62] | 9.53E-07 | 0.03832136 |
| rs7313078 | 12 | 128883721 | intron_variant | TMEM132C,  RP11-553N19.1 | 0.1415 | 0.01364 | 11.92 [3.37- 42.18] | 1.06E-06 | 0.03832136 |
| rs992586 | 7 | 14210392 | nc_transcript_variant | DGKB | 0.2593 | 0.05652 | 5.842 [2.884- 11.84] | 1.12E-06 | 0.03832136 |
| rs17003806 | X | 97535244 | intergenic_variant |  | 0.18 | 0.03097 | 6.868 [2.767- 17.05] | 1.12E-06 | 0.03832136 |
| rs41464146 | 13 | 47764748 | intergenic_variant |  | 0.1509 | 0.01802 | 9.689 [3.152- 29.78] | 1.14E-06 | 0.03832136 |
| rs41403245 | 8 | 52220546 | nc_transcript_variant | RP11-401H2.1 | 0.1481 | 0.01786 | 9.565 [3.114- 29.39] | 1.35E-06 | 0.04324526 |
| rs11177535 | 12 | 69575602 | intergenic_variant |  | 0.3889 | 0.1422 | 3.837 [2.249- 6.547] | 1.52E-06 | 0.0461782 |

**Additional file 1: Table S5**. Pairs of SNPs related to jPTC obtained by MDR analysis.

| **SNP 1** | **SNP 2** | **Gene 1** | **Gene 2** | **Gene name 1** | **Gene name 2** | **CVV** |
| --- | --- | --- | --- | --- | --- | --- |
| rs2574878 | rs17716031 | ENSG00000143799 | ENSG00000259560 | *PARP1* | *RP11-648K4.2* | 0.950 |
| rs2272407 | rs2287298 | ENSG00000065413 | ENSG00000070540 | *ANKRD44* | *WIPI1* | 0.942 |
| rs17716031 | rs9959080 | ENSG00000259560 | ENSG00000183287 | *RP11-648K4.2* | *CCBE1* | 0.941 |
| rs2354122 | rs4280318 | ENSG00000117419 | ENSG00000187323 | *ERI3* | *DCC* | 0.937 |
| rs17716031 | rs16959373 | ENSG00000259560 | ENSG00000140859 | *RP11-648K4.2* | *KIFC3* | 0.937 |
| rs17716031 | rs4251864 | ENSG00000259560 | ENSG00000011422 | *RP11-648K4.2* | *PLAUR* | 0.851 |
| rs1329985 | rs17716031 | ENSG00000223479 | ENSG00000259560 | *RP4-788P17.1* | *RP11-648K4.2* | 0.851 |
| rs17464981 | rs17716031 | ENSG00000183317 | ENSG00000259560 | *EPHA10* | *RP11-648K4.2* | 0.848 |
| rs17716031 | rs12479724 | ENSG00000259560 | ENSG00000089177 | *RP11-648K4.2* | *KIF16B* | 0.844 |
| rs17716031 | rs16943389 | ENSG00000259560 | ENSG00000140534 | *RP11-648K4.2* | *TICRR* | 0.844 |
| rs7168544 | rs17716031 | ENSG00000104043 | ENSG00000259560 | *ATP8B4* | *RP11-648K4.2* | 0.748 |
| rs6558073 | rs4280318 | ENSG00000012232 | ENSG00000187323 | *EXTL3* | *DCC* | 0.748 |
| rs17716031 | rs136866 | ENSG00000259560 | ENSG00000100150 | *RP11-648K4.2* | *DEPDC5* | 0.748 |
| rs17716031 | rs11650977 | ENSG00000259560 | ENSG00000174282 | *RP11-648K4.2* | *ZBTB4* | 0.747 |
| rs520088 | rs17716031 | ENSG00000090975 | ENSG00000259560 | *PITPNM2* | *RP11-648K4.2* | 0.747 |
| rs883201 | rs17716031 | ENSG00000171502 | ENSG00000259560 | *COL24A1* | *RP11-648K4.2* | 0.747 |
| rs16876356 | rs17716031 | ENSG00000132837 | ENSG00000259560 | *DMGDH* | *RP11-648K4.2* | 0.747 |
| rs17716031 | rs8132754 | ENSG00000259560 | ENSG00000160183 | *RP11-648K4.2* | *TMPRSS3* | 0.747 |
| rs6729766 | rs17716031 | ENSG00000237667 | ENSG00000259560 | *LINC01115* | *RP11-648K4.2* | 0.747 |
| rs3102477 | rs17716031 | ENSG00000253773 | ENSG00000259560 | *KB-1047C11.2* | *RP11-648K4.2* | 0.747 |
| rs6558073 | rs17716031 | ENSG00000012232 | ENSG00000259560 | *EXTL3* | *RP11-648K4.2* | 0.747 |
| rs17716031 | rs3736256 | ENSG00000259560 | ENSG00000251209 | *RP11-648K4.2* | *LINC00923* | 0.746 |
| rs11199560 | rs17716031 | ENSG00000227165 | ENSG00000259560 | *WDR11-AS1* | *RP11-648K4.2* | 0.746 |
| rs659092 | rs17716031 | ENSG00000255274 | ENSG00000259560 | *TMPRSS4-AS1* | *RP11-648K4.2* | 0.745 |
| rs11712216 | rs17716031 | ENSG00000163637 | ENSG00000259560 | *PRICKLE2* | *RP11-648K4.2* | 0.744 |
| rs10018612 | rs17716031 | ENSG00000072832 | ENSG00000259560 | *CRMP1* | *RP11-648K4.2* | 0.744 |
| rs9483919 | rs17716031 | ENSG00000146410 | ENSG00000259560 | *MTFR2* | *RP11-648K4.2* | 0.744 |
| rs3778307 | rs17716031 | ENSG00000135525 | ENSG00000259560 | *MAP7* | *RP11-648K4.2* | 0.744 |
| rs16928641 | rs17716031 | ENSG00000107731 | ENSG00000259560 | *UNC5B* | *RP11-648K4.2* | 0.744 |
| rs13401599 | rs17716031 | ENSG00000237667 | ENSG00000259560 | *LINC01115* | *RP11-648K4.2* | 0.744 |
| rs17716031 | rs11673276 | ENSG00000259560 | ENSG00000167633 | *RP11-648K4.2* | *KIR3DL1* | 0.744 |
| rs1945213 | rs17716031 | ENSG00000181395 | ENSG00000259560 | *OR5AL1* | *RP11-648K4.2* | 0.744 |
| rs4358155 | rs17716031 | ENSG00000237667 | ENSG00000259560 | *LINC01115* | *RP11-648K4.2* | 0.744 |
| rs10437600 | rs17716031 | ENSG00000133816 | ENSG00000259560 | *MICAL2* | *RP11-648K4.2* | 0.744 |
| rs4757260 | rs17716031 | ENSG00000133816 | ENSG00000259560 | *MICAL2* | *RP11-648K4.2* | 0.744 |
| rs16906616 | rs17716031 | ENSG00000254101 | ENSG00000259560 | *RP11-30J20.1* | *RP11-648K4.2* | 0.744 |
| rs4767020 | rs17716031 | ENSG00000089169 | ENSG00000259560 | *RPH3A* | *RP11-648K4.2* | 0.744 |
| rs6730761 | rs17716031 | ENSG00000157856 | ENSG00000259560 | *DRC1* | *RP11-648K4.2* | 0.744 |
| rs2274333 | rs17716031 | ENSG00000131686 | ENSG00000259560 | *CA6* | *RP11-648K4.2* | 0.744 |
| rs10775207 | rs17716031 | ENSG00000129038 | ENSG00000259560 | *LOXL1* | *RP11-648K4.2* | 0.743 |
| rs2659602 | rs17716031 | ENSG00000183715 | ENSG00000259560 | *OPCML* | *RP11-648K4.2* | 0.743 |
| rs11929494 | rs17716031 | ENSG00000196277 | ENSG00000259560 | *GRM7* | *RP11-648K4.2* | 0.740 |
| rs2272407 | rs1785819 | ENSG00000065413 | ENSG00000149256 | *ANKRD44* | *TENM4* | 0.658 |
| rs17716031 | rs733908 | ENSG00000259560 | ENSG00000100170 | *RP11-648K4.2* | *SLC5A1* | 0.654 |
| rs17075442 | rs17716031 | ENSG00000231894 | ENSG00000259560 | *WDR95P* | *RP11-648K4.2* | 0.653 |
| rs11129988 | rs17716031 | ENSG00000182983 | ENSG00000259560 | *ZNF662* | *RP11-648K4.2* | 0.653 |
| rs17716031 | rs8109103 | ENSG00000259560 | ENSG00000188227 | *RP11-648K4.2* | *ZNF793* | 0.653 |
| rs12475755 | rs17716031 | ENSG00000163053 | ENSG00000259560 | *SLC16A14* | *RP11-648K4.2* | 0.652 |
| rs10483420 | rs17716031 | ENSG00000151322 | ENSG00000259560 | *NPAS3* | *RP11-648K4.2* | 0.652 |
| rs2272407 | rs1317516 | ENSG00000065413 | ENSG00000240577 | *ANKRD44* | *RN7SL445P* | 0.652 |
| rs2272407 | rs7076530 | ENSG00000065413 | ENSG00000183621 | *ANKRD44* | *ZNF438* | 0.652 |
| rs2272407 | rs8132754 | ENSG00000065413 | ENSG00000160183 | *ANKRD44* | *TMPRSS3* | 0.652 |
| rs2272407 | rs7711859 | ENSG00000065413 | ENSG00000186952 | *ANKRD44* | *TMEM232* | 0.652 |
| rs2272407 | rs7757845 | ENSG00000065413 | ENSG00000080546 | *ANKRD44* | *SESN1* | 0.652 |
| rs2272407 | rs12540224 | ENSG00000065413 | ENSG00000048052 | *ANKRD44* | *HDAC9* | 0.652 |
| rs2272407 | rs12682345 | ENSG00000065413 | ENSG00000221914 | *ANKRD44* | *PPP2R2A* | 0.652 |
| rs2272407 | rs3788339 | ENSG00000065413 | ENSG00000100218 | *ANKRD44* | *RTDR1* | 0.652 |
| rs2272407 | rs9418829 | ENSG00000065413 | ENSG00000150760 | *ANKRD44* | *DOCK1* | 0.652 |
| rs17716031 | rs2294208 | ENSG00000259560 | ENSG00000100191 | *RP11-648K4.2* | *SLC5A4* | 0.652 |
| rs17716031 | rs1111034 | ENSG00000259560 | ENSG00000233324 | *RP11-648K4.2* | *EEF1A1P34* | 0.651 |
| rs1535392 | rs17716031 | ENSG00000082293 | ENSG00000259560 | *COL19A1* | *RP11-648K4.2* | 0.651 |
| rs9257403 | rs4280318 | ENSG00000204709 | ENSG00000187323 | *C6orf100* | *DCC* | 0.651 |
| rs17716031 | rs11648369 | ENSG00000259560 | ENSG00000260876 | *RP11-648K4.2* | *RP11-345M22.1* | 0.651 |
| rs1472493 | rs17716031 | ENSG00000151276 | ENSG00000259560 | *MAGI1* | *RP11-648K4.2* | 0.650 |
| rs17716031 | rs7055046 | ENSG00000259560 | ENSG00000102230 | *RP11-648K4.2* | *PCYT1B* | 0.650 |
| rs7512505 | rs17716031 | ENSG00000143799 | ENSG00000259560 | *PARP1* | *RP11-648K4.2* | 0.650 |
| rs10915848 | rs17716031 | ENSG00000143799 | ENSG00000259560 | *PARP1* | *RP11-648K4.2* | 0.650 |
| rs6816158 | rs17716031 | ENSG00000164035 | ENSG00000259560 | *EMCN* | *RP11-648K4.2* | 0.650 |
| rs11097740 | rs17716031 | ENSG00000164035 | ENSG00000259560 | *EMCN* | *RP11-648K4.2* | 0.650 |
| rs164495 | rs17716031 | ENSG00000013561 | ENSG00000259560 | *RNF14* | *RP11-648K4.2* | 0.650 |
| rs12580868 | rs17716031 | ENSG00000089154 | ENSG00000259560 | *GCN1L1* | *RP11-648K4.2* | 0.650 |
| rs17716031 | rs3108548 | ENSG00000259560 | ENSG00000186020 | *RP11-648K4.2* | *ZNF529* | 0.650 |
| rs932913 | rs17716031 | ENSG00000123171 | ENSG00000259560 | *CCDC70* | *RP11-648K4.2* | 0.650 |
| rs7039831 | rs17716031 | ENSG00000148219 | ENSG00000259560 | *ASTN2* | *RP11-648K4.2* | 0.650 |
| rs2186798 | rs17716031 | ENSG00000149294 | ENSG00000259560 | *NCAM1* | *RP11-648K4.2* | 0.650 |
| rs2057399 | rs17716031 | ENSG00000131018 | ENSG00000259560 | *SYNE1* | *RP11-648K4.2* | 0.650 |
| rs2517289 | rs17716031 | ENSG00000104760 | ENSG00000259560 | *FGL1* | *RP11-648K4.2* | 0.650 |
| rs12312562 | rs17716031 | ENSG00000089154 | ENSG00000259560 | *GCN1L1* | *RP11-648K4.2* | 0.650 |
| rs4598242 | rs17716031 | ENSG00000147485 | ENSG00000259560 | *PXDNL* | *RP11-648K4.2* | 0.650 |
| rs17716031 | rs7222554 | ENSG00000259560 | ENSG00000233852 | *RP11-648K4.2* | *AC005304.1* | 0.650 |
| rs17716031 | rs9896664 | ENSG00000259560 | ENSG00000180891 | *RP11-648K4.2* | *CUEDC1* | 0.650 |
| rs7441242 | rs17716031 | ENSG00000249460 | ENSG00000259560 | *RP11-665C14.2* | *RP11-648K4.2* | 0.650 |
| rs17716031 | rs9925929 | ENSG00000259560 | ENSG00000260876 | *RP11-648K4.2* | *RP11-345M22.1* | 0.650 |
| rs17716031 | rs3784873 | ENSG00000259560 | ENSG00000141012 | *RP11-648K4.2* | *GALNS* | 0.650 |
| rs7962663 | rs17716031 | ENSG00000111581 | ENSG00000259560 | *NUP107* | *RP11-648K4.2* | 0.649 |
| rs9844695 | rs17716031 | ENSG00000144644 | ENSG00000259560 | *GADL1* | *RP11-648K4.2* | 0.649 |
| rs17716031 | rs6614327 | ENSG00000259560 | ENSG00000179222 | *RP11-648K4.2* | *MAGED1* | 0.649 |
| rs16918062 | rs17716031 | ENSG00000253773 | ENSG00000259560 | *KB-1047C11.2* | *RP11-648K4.2* | 0.649 |
| rs2160543 | rs17716031 | ENSG00000048052 | ENSG00000259560 | *HDAC9* | *RP11-648K4.2* | 0.649 |
| rs13279773 | rs17716031 | ENSG00000147488 | ENSG00000259560 | *ST18* | *RP11-648K4.2* | 0.649 |
| rs9518820 | rs17716031 | ENSG00000238869 | ENSG00000259560 | *snoU13* | *RP11-648K4.2* | 0.649 |
| rs17716031 | rs6586246 | ENSG00000259560 | ENSG00000160183 | *RP11-648K4.2* | *TMPRSS3* | 0.649 |
| rs12293251 | rs17716031 | ENSG00000133816 | ENSG00000259560 | *MICAL2* | *RP11-648K4.2* | 0.649 |
| rs17396442 | rs17716031 | ENSG00000249816 | ENSG00000259560 | *LINC00964* | *RP11-648K4.2* | 0.649 |
| rs688019 | rs17716031 | ENSG00000151702 | ENSG00000259560 | *FLI1* | *RP11-648K4.2* | 0.649 |
| rs6802433 | rs17716031 | ENSG00000185008 | ENSG00000259560 | *ROBO2* | *RP11-648K4.2* | 0.646 |
| rs4547644 | rs17716031 | ENSG00000225742 | ENSG00000259560 | *RP11-513G11.4* | *RP11-648K4.2* | 0.646 |
| rs7181124 | rs17716031 | ENSG00000157890 | ENSG00000259560 | *MEGF11* | *RP11-648K4.2* | 0.561 |
| rs2272407 | rs4838592 | ENSG00000065413 | ENSG00000128805 | *ANKRD44* | *ARHGAP22* | 0.559 |
| rs2235544 | rs17716031 | ENSG00000211452 | ENSG00000259560 | *DIO1* | *RP11-648K4.2* | 0.559 |
| rs1939568 | rs17716031 | ENSG00000166004 | ENSG00000259560 | *KIAA1731* | *RP11-648K4.2* | 0.559 |
| rs8032721 | rs17716031 | ENSG00000198838 | ENSG00000259560 | *RYR3* | *RP11-648K4.2* | 0.559 |
| rs13248273 | rs17716031 | ENSG00000164751 | ENSG00000259560 | *PEX2* | *RP11-648K4.2* | 0.558 |
| rs12312562 | rs4280318 | ENSG00000089154 | ENSG00000187323 | *GCN1L1* | *DCC* | 0.558 |
| rs2272407 | rs6816158 | ENSG00000065413 | ENSG00000164035 | *ANKRD44* | *EMCN* | 0.558 |
| rs2272407 | rs1020995 | ENSG00000065413 | ENSG00000260661 | *ANKRD44* | *RP11-152L20.3* | 0.558 |
| rs2272407 | rs3785380 | ENSG00000065413 | ENSG00000166501 | *ANKRD44* | *PRKCB* | 0.558 |
| rs2272407 | rs10117186 | ENSG00000065413 | ENSG00000237372 | *ANKRD44* | *RP11-316P17.2* | 0.558 |
| rs2272407 | rs9918970 | ENSG00000065413 | ENSG00000106688 | *ANKRD44* | *SLC1A1* | 0.558 |
| rs2132662 | rs17716031 | ENSG00000137872 | ENSG00000259560 | *SEMA6D* | *RP11-648K4.2* | 0.558 |
| rs2480294 | rs17716031 | ENSG00000107951 | ENSG00000259560 | *MTPAP* | *RP11-648K4.2* | 0.558 |
| rs12385717 | rs17716031 | ENSG00000157933 | ENSG00000259560 | *SKI* | *RP11-648K4.2* | 0.557 |
| rs12781952 | rs17716031 | ENSG00000120549 | ENSG00000259560 | *KIAA1217* | *RP11-648K4.2* | 0.557 |
| rs9787172 | rs17716031 | ENSG00000184144 | ENSG00000259560 | *CNTN2* | *RP11-648K4.2* | 0.557 |
| rs502068 | rs17716031 | ENSG00000102683 | ENSG00000259560 | *SGCG* | *RP11-648K4.2* | 0.557 |
| rs896559 | rs17716031 | ENSG00000139174 | ENSG00000259560 | *PRICKLE1* | *RP11-648K4.2* | 0.557 |
| rs17716031 | rs7227537 | ENSG00000259560 | ENSG00000154655 | *RP11-648K4.2* | *L3MBTL4* | 0.556 |
| rs7780008 | rs17716031 | ENSG00000106617 | ENSG00000259560 | *PRKAG2* | *RP11-648K4.2* | 0.556 |
| rs17658052 | rs17716031 | ENSG00000130600 | ENSG00000259560 | *H19* | *RP11-648K4.2* | 0.556 |
| rs11059664 | rs17716031 | ENSG00000181234 | ENSG00000259560 | *TMEM132C* | *RP11-648K4.2* | 0.556 |
| rs4882749 | rs17716031 | ENSG00000181234 | ENSG00000259560 | *TMEM132C* | *RP11-648K4.2* | 0.556 |
| rs17610425 | rs17716031 | ENSG00000239498 | ENSG00000259560 | *AC114765.1* | *RP11-648K4.2* | 0.556 |
| rs540152 | rs17716031 | ENSG00000082293 | ENSG00000259560 | *COL19A1* | *RP11-648K4.2* | 0.555 |
| rs17716031 | rs284669 | ENSG00000259560 | ENSG00000124302 | *RP11-648K4.2* | *CHST8* | 0.555 |
| rs6758290 | rs17716031 | ENSG00000135973 | ENSG00000259560 | *GPR45* | *RP11-648K4.2* | 0.555 |
| rs17716031 | rs16948985 | ENSG00000259560 | ENSG00000260042 | *RP11-648K4.2* | *CTD-2034I21.1* | 0.555 |
| rs16837952 | rs17716031 | ENSG00000132694 | ENSG00000259560 | *ARHGEF11* | *RP11-648K4.2* | 0.555 |
| rs17716031 | rs12447241 | ENSG00000259560 | ENSG00000157423 | *RP11-648K4.2* | *HYDIN* | 0.555 |
| rs1147474 | rs17716031 | ENSG00000100626 | ENSG00000259560 | *GALNT16* | *RP11-648K4.2* | 0.555 |
| rs897328 | rs17716031 | ENSG00000100626 | ENSG00000259560 | *GALNT16* | *RP11-648K4.2* | 0.555 |
| rs1821625 | rs17716031 | ENSG00000176771 | ENSG00000259560 | *NCKAP5* | *RP11-648K4.2* | 0.555 |
| rs17392456 | rs17716031 | ENSG00000133808 | ENSG00000259560 | *MICALCL* | *RP11-648K4.2* | 0.555 |
| rs17716031 | rs7221379 | ENSG00000259560 | ENSG00000236770 | *RP11-648K4.2* | *AC079325.5* | 0.555 |
